# Babies Smell Wonderful to Their Parents, Teenagers Do Not: an Exploratory Questionnaire Study on Children’s Age and Personal Odor Ratings in a Polish Sample

**DOI:** 10.1007/s12078-017-9230-x

**Published:** 2017-06-27

**Authors:** Ilona Croy, Tomasz Frackowiak, Thomas Hummel, Agnieszka Sorokowska

**Affiliations:** 1 Department of Psychotherapy and Psychosomatic Medicine, TU Dresden, Fetscherstr. 74, 01307 Dresden, Germany; 2 Institute of Psychology, University of Wroclaw, ul. Dawida 1, 50-527 Wroclaw, Poland; 3 Smell & Taste Clinic, Department of Otorhinolaryngology, TU Dresden, Fetscherstr. 74, 01307 Dresden, Germany

**Keywords:** Body odor, Personal odor, Olfaction, Bonding, Child development

## Abstract

**Introduction:**

Infant body odor is subjectively pleasant to parents and activates reward areas in the brain. Hence, body odor perception might contribute to parental bonding. However, it is unknown whether the perceived pleasantness of children’s body odor varies over the course of a child’s development.

**Methods:**

Two hundred and thirty-five parents (*M* = 36.9 years, *SD* = 7.3) were asked to assess the personal odor pleasantness of their children (*N* = 367; *M* = 9.3 years, *SD* = 6.4).

**Results:**

Odor pleasantness was found to decrease as a function of children’s age. Neither sex of the parent nor sex of the child contributed significantly to this effect.

**Conclusions:**

We propose that the effect of age-related changes on personal odor pleasantness reflects olfactory modulation of parental-child relationships.

**Implications:**

Our study suggests that perception of young children’s personal odor as pleasant may contribute to bonding and thereby caretaking, which is needed to a lesser degree after puberty than before.

## Introduction

Parents typically report that the odor of their baby is one of the most pleasant scents that they can imagine. Women who were born without the sense of smell declare that they regret missing out on this seemingly wonderful experience (Bojanowski et al. 2013). Within days (Schaal et al. 1980), or even hours of giving birth, mothers can distinguish the smell of their child from the smell of other babies (Kaitz et al. 1987; Porter et al. 1983). Body odors of pre-pubertal children are evaluated as very pleasant in general (Ferdenzi et al. 2010; Fleming et al. 1993), and the sheer presence of a baby body odor activates reward-related brain areas (Lundstrom et al. 2013).

It seems that the perception of body odors may contribute to parent-child bonding. For example, the ability to recognize one’s own child fosters the expression of warm feelings toward the baby (Corter and Fleming 1995; Del Cerro 1998; Depue and Morrone-Strupinsky 2005). Affective bonds with the caregiver are essential for a child’s social and emotional development, and they are strongly dependent on initial caretaking (Bowlby 1988). Caretaking does, however, bear costs on the parents—in modern, Western societies, it is time-consuming and expensive. In addition, parents often prioritize the children’s needs above their own and the safety of the child becomes their major concern (Leckman et al. 1999). In the long term, caretaking is obviously rewarding, because it increases a person’s genetic fitness. However, this reward is rather implicit, in contrast to direct and immediate rewards associated with baby body odor (Lundstrom et al. 2013). Perceiving babies’ body odor as pleasant may thus be one of the mechanisms contributing to cost compensation and creation of familial bonds despite all problems associated with initial caretaking.

The parent-child relationship is affected and shaped by a multiplicity of changes during the development of the child, e.g., social or cognitive development. With increasing self-dependence, it is no longer pivotal that the parents always prioritize their child. Hence, the biological triggers of care, such as the baby schema (Kindchenschema) (Glocker et al. 2009), lose importance and vanish during transition into adulthood (Luo et al. 2011; Volk et al. 2007). We assume that the same is true for the child’s body odor. Anecdotal observations support this: While parents seem to enjoy the body odor of their babies, they rarely talk with the same fascination about the body odor of their pubertal or postpubertal children.

Body odors exhibit a considerable amount of variance, related to stable and variable compounds (Fialová et al. 2016; Leyden et al. 1981; Penn et al. 2007; Roberts et al. 2005; Schaefer et al. 2001). In a group of elements potentially important in the context of bonding, one can mention at least two different systems influencing body odor: peptides from the stable and genetically determined HLA complex (Milinski et al. 2013) and compounds related to development-dependent phenotypic traits (Thornhill et al. 2003). These developmental compounds change as a function of age and are related to the natural hormonal changes taking place as a child gets older (Blakemore et al. 2010).

It is thought that genetically determined body odor compounds (resulting from the genetic similarity of children and their parents) foster initial bonding. The sense of smell is known to be involved in kin recognition in animals (e.g., hamsters (Todrank et al. 1998)) and in humans (Havlicek and Roberts 2009). Kin recognition fulfills two critical biological functions. First, it facilitates attachment to family members which in turn increases investment in genetically related individuals. Accordingly, altruistic tendencies toward genetic relatives are higher than those directed toward unrelated individuals in many animal species including humans (Burnstein et al. 1994; Chapais et al. 2001). Second, kin recognition facilitates inbreeding avoidance, wherein inbreeding is associated with reduced fitness of resultant offspring (Park et al. 2008); mating of genetically similar partners may lead to inbreeding depression, i.e., higher rates of dangerous mutations (Charlesworth and Charlesworth 1987). In support of this, it has been found that couples who cohabitated early in childhood have an increased sexual aversion toward one another (Wolf 1995). It is thus possible that kin recognition depends not only on genetic factors, but that it can also be learned through exposure during childhood.

The interaction between genetic and developmental factors during the course of a child’s growth might serve as an incest-avoidance barrier. Two existing studies on this issue (Ferdenzi et al. 2010; Weisfeld et al. 2003) report contradictory results on whether parents of pubertal children find the odors of their children pleasant. Weisfeld and collaborators (Weisfeld et al. 2003) found that body odors of pubertal children were perceived as unpleasant by the parents. However, Ferdenzi et al. (2010) did not observe this effect. Both of these studies have the strong advantage of utilizing real body odor samples. However, because it is difficult to investigate body odor perception within families, these studies were conducted in small sample sizes of 24 (Weisfeld et al. 2003), and 18 children (Ferdenzi et al. 2010) in total. Unfortunately, this prevents us to draw definite conclusions about age-related changes in body odor pleasantness, and more data are clearly warranted. We used a less costly method using a questionnaire on a larger sample of 367 children; we tested whether the perceived pleasantness of a child’s personal odor decreases as a function of age. In addition, we tested whether a potential drop in personal odor pleasantness affected opposite-sex parent-child relationships more than same-sex parent child relationships. Different patterns of changes in pleasantness for same/opposite-sex parents would address the hypotheses concerning inbreeding avoidance. Generally, potential incest might be problematic in relationships with opposite-sex parents. Therefore, if smell-based inbreeding avoidance was observed, personal odor of own pubertal and postpubertal children should be less pleasant for opposite-sex parents than for same-sex parents.

## Method

### Participants

Our study originally comprised 283 parents. None of those parents had shared offspring, so either father or mother of a family was included in the study. All of them spoke Polish. The participating parents were recruited from various university campuses across Wroclaw and Brzeg (Poland). They were students, participants of vocational courses, participants of local sports events organized at the campuses, participants of evening courses for adults, and passersby (the campuses are located close to the city center). They were approached by the experimenters and trained research assistants and asked to complete a short questionnaire. The paper-and-pencil questionnaires were then individually completed by people who declared having at least one child. Participants were asked (but not formally tested) whether they experience any olfactory problems (i.e., whether they assess their smell as poor or if they had any chronic diseases associated with nose or sinuses). Three parents declared to have problems with their sense of smell and were excluded from the final sample. We also excluded all parents who declared that they were unable to recognize the body odor of their children (*n* = 20), nonbiological parents (*n* = 10), and people who declared that they spent less than 10 min per day with their children (*n* = 7).

The final sample included 235 parents (163 mothers and 72 fathers), aged between 21 and 65 years (*M* = 36.9 years, *SD* = 7.3). Of these, 28 were single parents, 206 were in a relationship, and one did not indicate an answer to this question. The participants were parents to 367 children aged from 1 month to 35 years (*M* = 9.3 years, *SD* = 6.4). Of these, 184 were girls and 183 were boys. On average, the parents were 27.0 ± 5.2 years older than their first child. There was a small negative correlation between the children’s age and the parent-child age difference (*r* = −0.22, *p* = 0.001), reflecting the current sociodemographic trend of having children at an increasingly older age.

For the purpose of further analyses, the children were divided into four age groups according to the main stages of hormonal development (Dorn et al. 2006): less than 4 years (infants), 4 to 8 years (prepubertal), 9 to 14 years (pubertal), and more than 14 years old (postpubertal). Grouping was based on age, not on the individual development of the children. Table 1 presents descriptive statistics for each age group.

**Table 1:** Descriptive statistics for each of the age groups of children

		Age category											
		<4 years *N* = 79			4 to 8 years *N* = 114			9 to 14 years *N* = 68			>14 years *N* = 105		
		*M*	*SD*	*n*	*M*	*SD*	*n*	*M*	*SD*	*n*	*M*	*SD*	*n*
Age (years)		1.9	1.0		5.8	1.4		10.6	1.4		17.8	3.5	
Parent-child relationship		18.6	1.7		18.5	1.6		17.8	2.5		17.0	2.9	
Personal odor pleasantness		18.2	2.4		17.4	2.8		16.6	3.5		15.2	4.0	
Sex of child	Female			39			56			31			57
	Male			40			58			37			48

All subjects provided written informed consent prior to their inclusion in the study. The study was approved by the ethical board of the Institute of Psychology (University of Wroclaw, Poland), and it was carried out in accordance with the provisions of the World Medical Association Declaration of Helsinki.

### Procedure

The parents completed a questionnaire containing questions related to their current partner and children. In the first part of the questionnaire, the participants assessed their relationship with a current partner (visual analogue scale with anchors “very bad” and “very good” as extremes and “neutral” in the middle). They also declared whether they knew their partner’s natural body odor, and when they did, they rated the pleasantness of this odor on a visual analogue scale with anchors “very unpleasant” and “very pleasant” as extremes and neutral in the middle (1.8% who did not know the odor of their partner, and 12.4% who did not answer this question were excluded from the analyses). In the second part of the questionnaire, the parents declared how old their “Child 1” was and marked the child’s sex and whether the child was their biological child. Further, the parents were asked “How much time do you spend with the child during an average working day?” and “How much time do you spend with the child during an average weekend day?” (several hours per day, about 1 h per day, about 10 min per day, less than 10 min per day). Finally, similar to the assessments regarding their partner, the parents assessed their relationship with the child and they rated the pleasantness of the child’s body odor. When the participant had more than one child, he or she was given separate questionnaire sheets for each consecutive child. The answers were coded on a 0–20-point scale, with higher numbers corresponding to a relatively better reported relationship and to higher personal odor pleasantness.

### Statistical Methods

Data were analyzed using SPSS 21 in several steps. Data inspection showed that one parent rated the body of the child by far less pleasant than the rest of the parents (about 4 SDs below the group mean). Robust covariance was chosen in the further model to deal with this potential outlier.

First, single linear regression analyses were used to identify potential confounds in parents’ ratings of personal odor pleasantness. Sex of the children and parents, time spent together on an average working day, and satisfaction with the relationship with the child were used as predictors for the target (rating of child personal odor pleasantness). Results showed that satisfaction with the relationship (*F*[1,365] = 49.1, *p* < 0.001; *β* = 0.38) and time spent together (*F*[1,365] = 9.6, *p* < 0.00; *β* = 0.25) significantly impacted the personal odor rating. There were no other significant effects. As both variables correlated with the age of the child (relationship satisfaction: *r* = −0.29, *p* < 0.001; time spent together: *r* = −0.25, *p* < 0.001), they were added as confounders for further analysis.

Second, the data were analyzed using a generalized mixed model. Each parent served as subject, and multiple children per parent were treated as repeated measurements. The following fixed main effects were included: age of the child as continuous variable (not grouped), sex of the parent, sex of the child, and the interaction sex of parent by sex of child. Time spent together and overall relationship satisfaction were included as random effects. Robust estimation of covariance was used for calculation. For further analysis and purpose of visualization, children were grouped according to age (see Table 1).

To investigate the interaction between parent’s and child’s sex (potential effect of incest avoidance), the rating data were grouped by same-sex (mother-daughter; father-son) and opposite-sex (mother-son, father-daughter), and were analyzed using the generalized mixed model approach with same-opposite sex and age group as main factors and age group by same-opposite sex as an interaction factor. Time spent together and overall parent-child relationship satisfaction were included as random effects. Robust estimation of covariance was used for calculation. For both generalized mixed model analyses, we also checked the impact of the order of children. For this purpose, we included another random effect in the analysis which indicated for each child whether it was the first, second, or third child in a given family. This did not change the results.

For the purpose of data visualization, personal odor ratings were grouped into the following categories: very unpleasant (0–4), unpleasant (4–8), neutral (8–12), pleasant (12–16), and very pleasant (16–20).

## Results

**Fig. 1:**
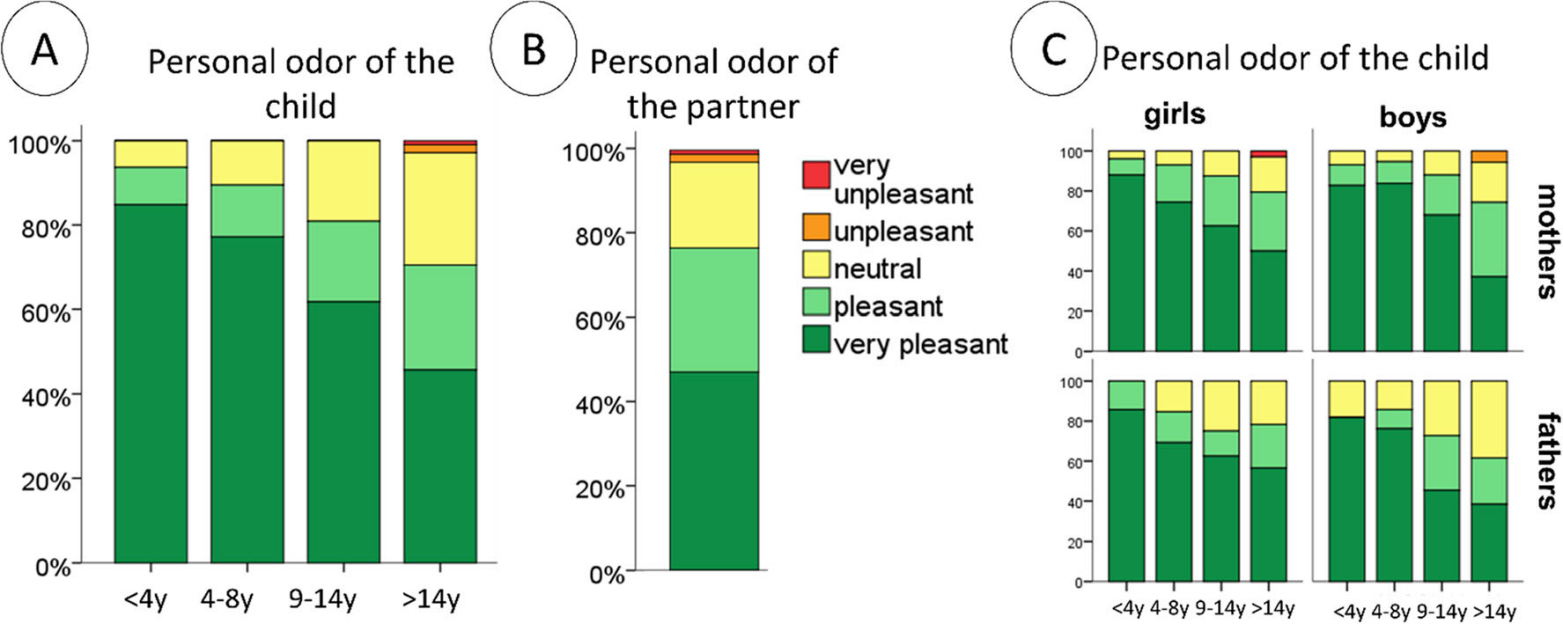
**a** Personal odor pleasantness in relation to the age of the child. A significant decrease was observed as a function of children’s age. Personal odor ratings for the oldest children were approximately in the same range as those for partners (**b**). Ratings for same-sex and opposite-sex children follow a similar pattern (**c**). For visualization purposes, all personal odor ratings were grouped into the following categories: very unpleasant (0–4), unpleasant (4–8), neutral (8–12), pleasant (12–16), and very pleasant (16–20)

Age of the child significantly impacted on ratings of personal odor pleasantness (*F*[1,361] = 16.2, *p* < 0.001). Ratings of personal odor pleasantness decreased as a function of the child’s age (compare Table 1 and Fig. 1). Sex of the parents did not contribute significantly (*F*[1,361] = 0.49, *p* = 0.49), and there was no significant effect of sex of child (*F*[1,361] = 0.02, *p* = 0.89) or an interaction between sex of parent and child (*F*[1,361] = 0.4, *p* = 0.53) on the perceived personal odor pleasantness (compare Table 2 and Fig. 1). The same pattern of results was found when the data were grouped into age categories. Thus, age category significantly affected ratings of personal odor pleasantness (*F*[3,359] = 7.8, *p* < 0.001) and perceived personal odor pleasantness decreased linearly as a function of a child’s age (Fig. 1). There was no effect of the sex of the parent (*F*[1,359] = 0.59, *p* = 0.44) or child (*F*[1,359] = 0.06, *p* = 0.81) and no interaction between sex of parent and child (*F*[1,359] = 0.37, *p* = 0.54).

**Table 2:** Personal odor pleasantness rating according to sex of the parent and child

		Sex of children																							
		Girls												Boys											
		Age of children												Age of children											
		<4 years			4 to 8 years		9 to 14 years				>14 years			<4 years			4 to 8 years		9 to 14 years				>14 years		
		*M*	*SD*	*n*	*M*	*SD*	*n*	*M*	*SD*	*n*	*M*	*SD*	*n*	*M*	*SD*	*n*	*M*	*SD*	*n*	*M*	*SD*	*n*	*M*	*SD*	*n*
Sex of parents	Mothers	18.6	2.2	25	17.3	2.8	43	16.5	3.3	24	15.7	4.4	34	17.9	2.5	29	17.8	2.3	37	17.2	3.2	25	14.6	3.8	35
	Fathers	18.4	1.6	14	16.8	3.6	13	16.4	4.2	8	15.7	3.8	23	17.6	3.7	11	17.2	3.4	21	15.6	3.9	12	14.3	3.9	13

Planned post hoc tests revealed that perceived personal odor pleasantness was significantly higher for infants than for pubertal (*t* = 2.5, *p* = 0.02) and postpubertal (*t* = 4.6, *p* < 0.001) children. Figure 1 shows that for infants, 93.7% of the parents rated their child’s personal odor as pleasant or very pleasant; for pubertal children, this proportion dropped to 83.8%, and for postpubertal children, only 75.2% of parents rated the personal odor of their child as a pleasant sensation. This latter value matches the average rating given for a partner’s personal odor, which was rated as pleasant or very pleasant by 76.7% of the participants.

As indicated in the “Statistical Methods” section, we decided to investigate the interaction between parent’s and child’s sex to observe whether there was a potential effect of incest avoidance in our sample. There was no significant overall effect of same-opposite sex (*F*[1,358] = 0.4; *p* = 0.5), and importantly, there was no significant interaction between same-opposite sex and age group (*F*[1,358] = 0.5, *p* = 0.7).

## Discussion

In line with both, our prediction and previous findings (Ferdenzi et al. 2010; Fleming et al. 1993), our study showed that parents very much like the personal odor of their young children. However, the older the child, the more the personal odor ratings of parents dropped. Personal odor of postpubertal children, although still rated as positive (above neutral), was significantly less pleasant than that of infants.

It is possible that developmental changes in children’s hormone levels modulate parents’ perceptions of their personal odors. Increases in sex hormone levels around the time of puberty may modulate personal odors in such a way that they are rated less favorably by parents. Consequently, the initially very high pleasantness ratings of a child’s personal odor decrease in postpubertal children to approximately the level of how personal odors of other beloved people (e.g., the partner) are rated.

In principle, two main stages of hormonal development can be distinguished (Dorn et al. 2006): adrenarche and gonadarche. Adrenarche, or activation of the hypothalamic-pituitary-adrenal (HPA) axis, typically begins between the age of 6 and 9 in girls and a year later in boys. Adrenal androgens begin to rise and contribute to the development of secondary sexual characteristics such as axillary and pubic hair and changes in body odor. Gonadarche begins with activation of the hypothalamic-pituitary-gonadal (HPG) axis and ends with the attainment of fecundity. The maturing ovaries and testes also secrete the gonadal steroids estrogen and testosterone, respectively. Derivates of those hormones change body odor throughout a child’s development (Chopra et al. 2008). This process terminates around the age of 8 to 14 years in girls and 9 to 15 in boys. Interestingly, the earlier onset of developmental hormones in girls was reflected in our data (see Fig. 1c), although not statistically significant.

We did not find support for a potential incest avoidance effect on perceived odor pleasantness, as the effect of enhanced body odor aversions in opposite-sex parent-child relations (Weisfeld et al. 2003) could not be confirmed. In line with previous suggestions (Lieberman et al. 2003), it seems that olfactory recognition mechanisms are far more likely to support close parent-child bonding before sexual maturity than inbreeding avoidance after maturity. However, further studies in this area are necessary before any definite conclusions can be made.

We are aware of several limitations of the study. First, personal odor pleasantness was retrieved from memory. Although we controlled for potential biases, such as reduced olfactory function and/or memory of the child’s personal odor, other potential confounds such as sinunasal diseases could not be entirely eliminated. Also, the reports of perceived pleasantness could be driven by other factors than a child’s natural body odor. Various cultural practices contribute to the final personal odor (Allen et al. 2015), and we can expect that their influence becomes stronger with age as pubescents/adolescents begin to smoke, drink, wear perfume, etc., hence differentially affecting parental reports across the age categories (Martinec Nováková et al. 2017). Further, parents’ ratings were not blind to the identity of a rated person and as a result could be superimposed by a general evaluation of the child, which is also reflected in the rather high correlation between odor pleasantness and relationship satisfaction. Although we controlled for parent-child relationship ratings in our analyses, we cannot exclude the possibility that some results were partly due to a so-called “halo” effect (Nisbett and Wilson 1977). Especially as our data were collected via questionnaires and not in an experimental setting, such effects are possible.

Our sample was based on volunteers; therefore, a participation bias may occur. It has for instance been shown that volunteers are more likely to be female, highly educated. and extraverted compared to nonvolunteers of experimental studies (Bortz and Döring 2013). Because our entire sample was recruited in one country, we cannot generalize for different countries or cultures. Finally, subtle effects such as potential incest avoidance are unlikely to be fully reflected in our data and might be better assessed using a blinded procedure of body odor assessment.

Despite these limitations, our study provides novel support for a hypothesis that body odor perception plays a potentially strong role in parent-child relationship. It further suggests that research involving children’s personal odors should not collapse across age, but include age or at least age group as suggested in our study, as a moderating factor.
